# Characterizing Disease Progression in Parkinson’s Disease from Videos of the Finger Tapping Test

**DOI:** 10.1109/TNSRE.2024.3416446

**Published:** 2024-06-26

**Authors:** Diego L. Guarín, Joshua K. Wong, Nikolaus R. McFarland, Adolfo Ramirez-Zamora

**Affiliations:** Department of Applied Physiology and Kinesiology, University of Florida, Gainesville, FL 32607 USA; Department of Neurology and the Norman Fixel Institute for Neurological Diseases, University of Florida, Gainesville, FL 32607 USA.; Department of Neurology and the Norman Fixel Institute for Neurological Diseases, University of Florida, Gainesville, FL 32607 USA.; Department of Neurology and the Norman Fixel Institute for Neurological Diseases, University of Florida, Gainesville, FL 32607 USA.

**Keywords:** Parkinson’s disease, pose estimation, video signal processing

## Abstract

**Introduction:**

Parkinson’s disease (PD) is characterized by motor symptoms whose progression is typically assessed using clinical scales, namely the Movement Disorder Society-Unified Parkinson’s Disease Rating Scale (MDS-UPDRS). Despite its reliability, the scale is bounded by a 5-point scale that limits its ability to track subtle changes in disease progression and is prone to subjective interpretations. We aimed to develop an automated system to objectively quantify motor symptoms in PD using Machine Learning (ML) algorithms to analyze videos and capture nuanced features of disease progression.

**Methods:**

We analyzed videos of the Finger Tapping test, a component of the MDS-UPDRS, from 24 healthy controls and 66 PD patients using ML algorithms for hand pose estimation. We computed multiple movement features related to bradykinesia from videos and employed a novel tiered classification approach to predict disease severity that employed different features according to severity. We compared our video-based disease severity prediction approach against other approaches recently introduced in the literature.

**Results:**

Traditional kinematics features such as amplitude and velocity changed linearly with disease severity, while other non-traditional features displayed non-linear trends. The proposed disease severity prediction approach demonstrated superior accuracy in detecting PD and distinguishing between different levels of disease severity when compared to existing approaches.

## I. Introduction

Parkinson’s disease (PD) is a multi-symptomatic neurodegenerative disorder for which disease-modifying or preventive therapies are not currently available. Current therapies focus on mitigating symptoms such as bradykinesia, tremor, postural instability, and mood disturbance. There is great interest in developing novel therapies that can slow down disease progression [1].

The Movement Disorder Society – Unified Parkinson’s Disease Rating Scale (MDS-UPDRS) is one of the most used outcome measures to assess disease progression, severity, and the effect of therapies [2], [3]. The MDS-UPDRS Part III offers high test-retest reliability and is a sensitive measure of disease progression in the mild, moderate, and late stages of disease [4], [5]. However, MDS-UPDRS Part III scoring is bound by broad definitions that are anchored to a 5-point Likert scale that has limited resolution in characterizing motor symptoms and is prone to subjective interpretation [6], [7]. Further, as this scale is based on symptomatic parkinsonism, it has limited sensitivity to detect the early or prodromal state of PD. Clinical assessment in PD would unquestionably benefit from an outcome measure that is straightforward, objective, reliably measures motor function, and is sensitive to small changes in disease progression. Such an outcome measure would ensure consistent measures across different disease severities and conditions.

Recently, there has been a growing interest in establishing objective digital movement markers of PD based on video recordings of the MDS-UPDRS III using machine learning (ML) algorithms for pose estimation. In particular, the Finger Tapping test, a component of the MDS-UPDRS III used to asses upper limbs bradykinesia, has gained significant attention [8], [9], [10], [11]. Several research groups, including ours, have developed custom processing pipelines to extract objective movement features from videos of the Finger Tapping test using ML algorithms. Such pipelines have been used to detect PD from videos with accuracy higher than 80% [11], predict disease severity [8], [9], and quantify the effect of therapies such as deep brain stimulation [10]. Assessing PD motor symptoms severity through objective kinematic features derived from videos of the MDS-UPDRS III benefits from the scale’s established clinical validity and widespread acceptance in the medical community while avoiding the limitations of the 5-point Likert scale.

Current approaches that use videos and machine learning algorithms to evaluate PD severity rely on a common set of kinematic features across disease severity, with the assumption that these features vary consistently with disease severity. However, this assumption may not hold true since motor symptoms do not necessarily change uniformly as PD progresses. We hypothesize that different kinematic features may become more significant and reliable at detecting PD and predicting motor symptoms severity at different stages of PD.

In this study, we analyzed video data from 24 healthy controls and 66 persons with PD. We used ML algorithms for hand pose estimation to compute video-based kinematic features related to bradykinesia. Using these features, we tested our hypothesis with two different approaches. First, we tested three classification approaches for prediction of disease severity based on kinematic features captured by video. These approaches included a) a multiclass classification model that uses a consistent set of features for all severity levels, b) an ordinal binary classification approach that reflects the ordinal nature of disease severity scores, and c) a novel tiered binary classification approach that employs different movement features according to disease severity. Second, we conducted statistical analyses to identify which kinematic features differ between healthy controls and persons with PD, and how these features changed as a function of disease severity.

## II. Methods

### A. Data Acquisition

Videos from 66 participants with PD and 24 age matched healthy controls were used in this study. The data and acquisition protocol are described in detail elsewhere [12]. Briefly, participants with PD (<5 years of diagnosis) with a diagnosis confirmed by a movement disorders specialist using the UK PD Brain Bank diagnostic criteria [13] were recruited. All study-related data acquisition sessions occurred at UF Health. Participants were ineligible if they had a prior history of stroke or brain tumor, had an implanted electrical device (cardiac pacemaker or neurostimulator) or aneurysm clip, or were pregnant or nursing.

Participants with PD who met all eligibility criteria participated in two data acquisition sessions, including a baseline session and a 1-year follow-up session. Data acquisition sessions were performed in the OFF-medication state after overnight withdrawal from antiparkinsonian medications, and included motor and cognitive assessments, such as the MDS-UPDRS III. PD subjects were enrolled in a clinical trial to evaluate the effect of rasagiline in PD. There was no statistically significant difference between the baseline and 1-year follow-up sessions for all the analyzed outcome measures, including the MDS-UPDRS III [12]. Consequently, treatment arm was not considered as a variable in this study. Healthy controls were recruited from the community; those who met the eligibility criteria performed a single baseline test consisting of secondary motor and cognitive testing, including the MDS-UPDRS-III.

**Fig. 1.**
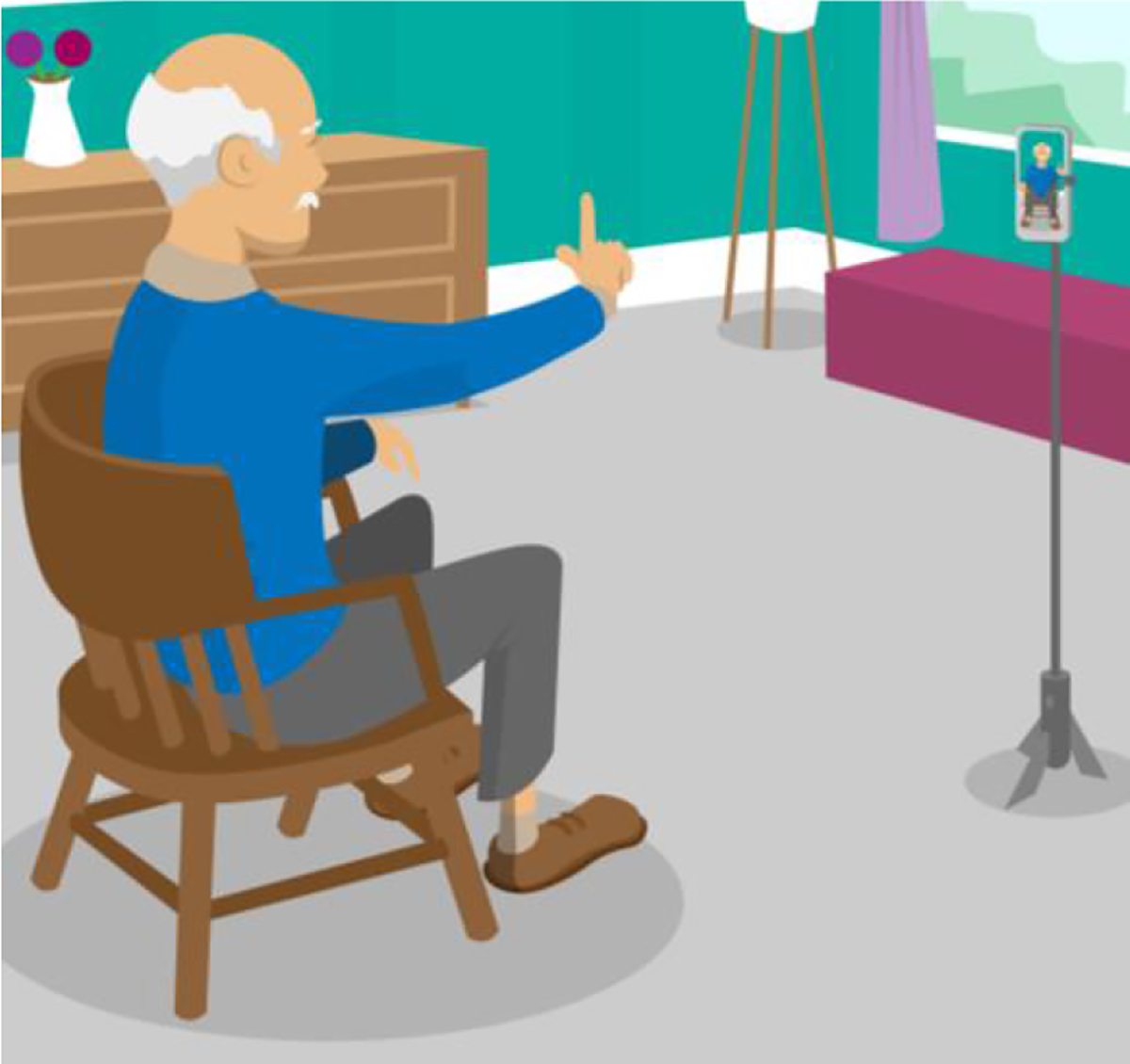
Recording set-up and environment. Subjects sit in front of a standard video camera and perform the Finger Tapping task. The task is recorded, and the video is stored for processing. The task performance is guided by an expert clinician who provides a clinical score.

MDS-UPDRS III assessments were video recorded. Fig. 1 shows a schematic representation of the recording set-up and environment. Subject sat comfortably on a chair and videos were recorded using a standard video camera mounted on a tripod. Standard, RGB videos were recorded at 30 FPS with a resolution of 1920×1080 pixels. Video were stored in a local server and later accessed for processing.

During the execution of the MDS-UPDRS III, a trained clinician stood in front to the participant and provided clear instructions on how to perform each task. The clinician evaluated the performance using a pre-defined criteria [2]. Tasks were manually evaluated using a severity score from 0 to 4, where 0 indicates normal movement, and scores of 1 to 4 indicate slight, mild, moderate, and severe motor symptoms severity respectively.

Videos were recorded for all healthy participants. As for the subjects with PD, some had their videos recorded only once, either during the initial baseline evaluation or at the 1-year follow-up visit, whereas others had recordings made at both visits. To avoid using repeated measures for only a few subjects, our study used data from healthy controls, PD subjects with only one video recording (either baseline or 1-year follow-up visits), and the baseline recording of persons with PD who have two recordings.

Participants gave informed consent to take part in this study following the Declaration of Helsinki. The University of Florida Institutional Review Boards approved this study.

### B. Video Processing

**Fig. 2.**
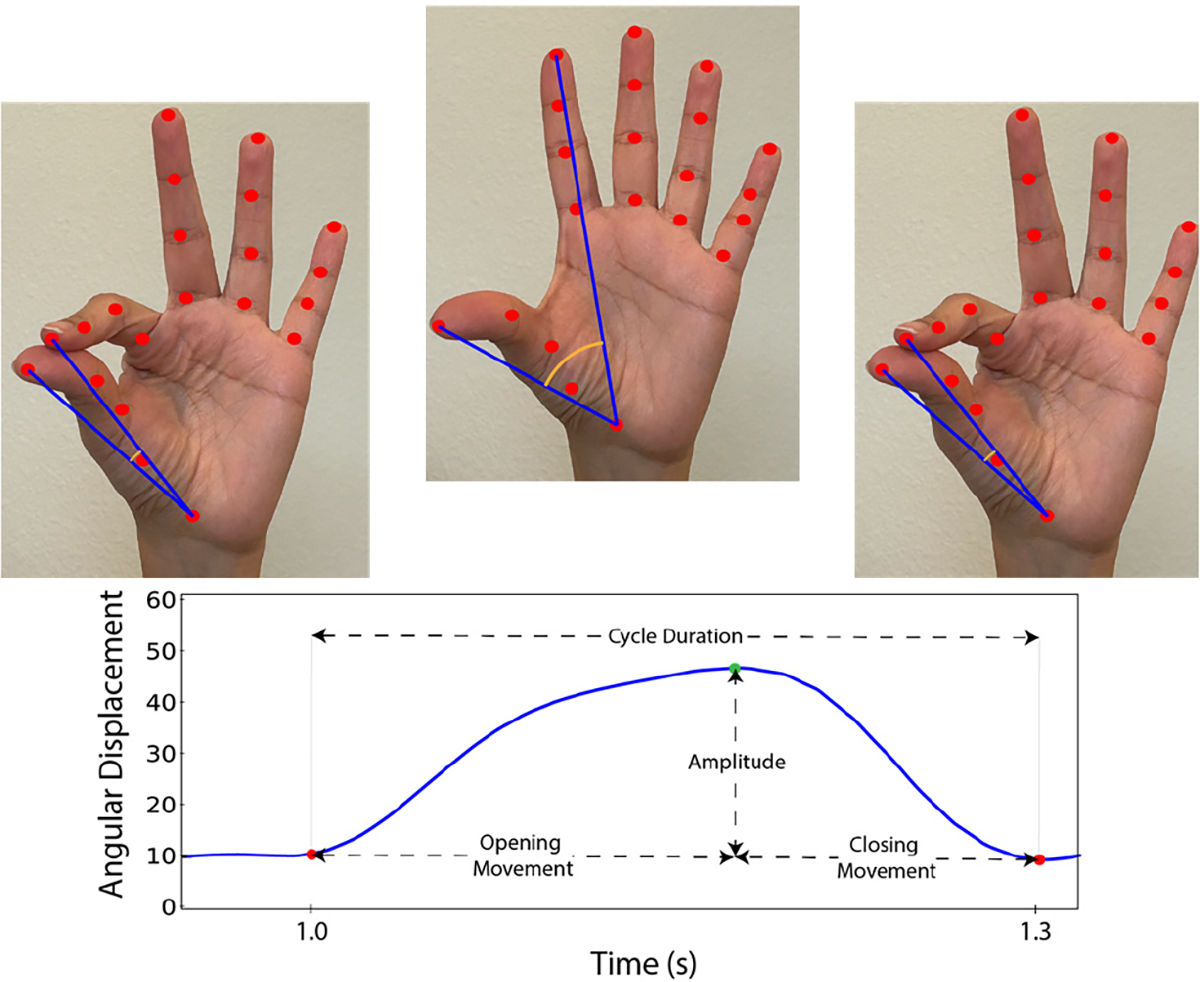
Hand tracking results provided by our video processing pipeline. We compute the angular distance between two vectors formed by joining the base of the hand with the tip of the index and thumb fingers as localized by Google’s MediaPipe in each video frame. The angular distance is tracked through the video to estimate an angular displacement signal. The bradykinesia related kinematic features are then computed from the peaks and valleys (green and red dots) of the angular displacement signal.

MDS-UPDRS III videos were manually processed to identify the start and end time of the Finger Tapping test for the right and left hands. Finger Tapping videos were processed using our custom pipeline [10]. As Fig. 2 shows, we used Google’s MediaPipe markerless hand pose estimation and tracking algorithm to localize the hands in the video and estimate the position of 21 hand landmarks in each video frame [14]. The landmarks consist of the hand’s base and four points on each finger, including the fingertips and joints. We have previously validated the accuracy of this algorithm in persons with PD [10]. Using the landmarks, we calculated the angular separation between the thumb and index finger by computing the angle between two vectors formed by connecting the finger’s tips with the hand’s base.

Fig. 2 shows the angular displacement signals obtained after low pass filtering the raw angular separation data (cut-off frequency of 7Hz). The signals’ peaks and valleys were automatically estimated using standard algorithms. The signal’s peaks and valleys correspond to the maximum opening and closing of the fingers.

### C. Extraction of Kinematic Features

Fig. 2 demonstrates the kinematic features extracted from the movement displacement signal. In particular, we computed the mean and coefficient of variation (CV) of the movement amplitude, the mean and CV of the movement speed (movement amplitude / movement duration), the mean and CV of the opening movement speed (movement amplitude/opening movement duration), the mean and CV of the closing movement speed (movement amplitude/ closing movement duration), the mean, CV, and range of the cycle duration, the movement rate (number of taps over time), and the amplitude decay (the mean amplitude during the first half of the trial over the mean amplitude during the second half of the trial).

### D. Data Analysis

We analyzed the data to determine the effectiveness of using kinematic features from Finger Tapping test videos to predict the severity of motor signs in PD as assessed by clinicians. We compared three different classification approaches, including a multiclass classification model [15], [16], [17], [18], [19], an ordinal binary classification approach [8], and our new tiered binary classification approach.

**Fig. 3.**
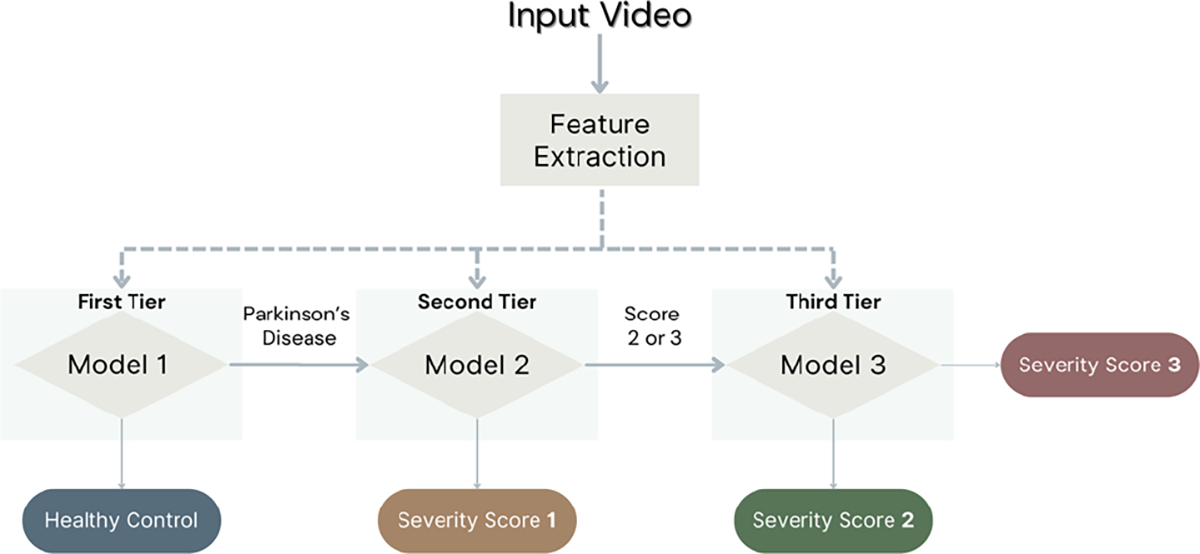
Graphical description of the proposed Tiered Binary Classification approach. A set of features is extracted from the input video using ML algorithms for hand pose estimation. Then three binary classification models are trained to identify different severity levels. Model 1 identifies if the video belongs to a Healthy Control or a person with PD. If the video belongs to a person with PD, Model 2 is used to identify if the video should receive a severity score of 1 or higher. If the video should receive a severity score higher than 1, Model 3 is used to identify if the video should receive a score of 2 or 3. Each model uses different movement features tailored to the disease severity.

1. *Multiclass Classification:* In this approach, a single multiclass classification model is trained to classify all motor symptoms severities based on video-based kinematic features. That is, the model output corresponds to the probability that a Finger tapping video is assigned a severity score of 0, 1, 2, or 3.
2. *Ordinal Binary Classification:* This approach was originally introduced by Morinan et at. [8]. In this approach, multiple binary classification models are trained to identify the motor symptoms severity based on video-based kinematic features. There are a total of three binary classification models. The first model classifies videos with a score of 0 against scores 1, 2, or 3; the second model classifies videos with a scores 0 or 1 against scores 2 or 3; and the third model classifies videos with a score of 0, 1, or 2 against scores 3.
3. *Tiered Binary Classification:* In this approach, multiple binary classification models are trained to identify the motor symptoms severity based on video-based kinematic features. Fig. 3 present a graphical description of the proposed approach. There are a total of three binary classification models, each model is designed to identify different levels of disease severity. That is, the first model classifies healthy controls from persons with PD. If the first model indicates that the video belongs to a person with PD, then the second model classifies scores of 1 against scores 2 or 3. Finally, if the second model indicates that the video should receive a score higher than 1, the third model classifies scores of 2 against scores 3.

Classification was achieved using a logistic regression model for classification. All models included the video derived kinematic features described above along with the subject’s age and sex as classification features. Features were normalized to have zero mean and unit standard deviation before model training. To mitigate the risk of overfitting, we used a recursive feature elimination (RFE) method, which systematically removes the least influential features—those with minimal impact on the model’s coefficients. After removing a non-influential feature, the model is retrained with the remaining features. This iterative process is repeated until any further reduction in features no longer yielded an improvement in model performance [20].

Data was split into training and testing sets, with 70% of the data used for model training and 30% for model testing. The logistic regression models were trained using a five-fold cross-validation approach using only the training data. The results presented in this manuscript correspond to those obtained for the testing data. Classification models were compared based on the Area Under Curve (AUC) of the Recall-Precision curve and the f1-score. Precision refers to the ratio between the true positives and the sum of true positives and false positives, and recall refers to the ratio between the true positives and the sum of true positives and false negatives. The f1-score is the harmonic mean of precision and recall. We choose these measurements as they better represent the model performance for imbalanced datasets. All the analysis was performed using the scikit-learn library for Python [21].

As our data are unbalanced, we used the Synthetic Minority Oversampling Technique (SMOTE) algorithm to oversample the under-represented classes and under-sample the overly-represented classes in our data to produce datasets with balanced classes [22]. This technique is commonly used in clinical application were unbalanced datasets are common [8], [17], [23], [24], [25].

Finally, we conducted statistical tests to determine whether the movement features showed significant differences between clinician assigned scores (0, 1, 2, 3, and 4). Differences between groups were evaluated using ANOVA for normally distributed data and Welch ANOVA for non-normally distributed data. To assess normality of our data, we employed the one-sample Kolmogorov–Smirnov test. For groups that demonstrated significant difference, we then performed a Tukey’s Honest Significant Difference post-hoc test with Bonferroni correction to identify which groups were significantly different. A p-value < 0.05 was considered to indicate a statistically significant difference. All statistical analyses were performed using the SciPy and Pingouin Libraries for Python [26], [27].

## III. Results

### A. Data

TABLE I summarizes the demographic information for the study’s participants. The data included a total of 180 videos, 44 from healthy individuals and 123 from persons with PD. There were 42 videos with a clinician provided motor symptoms severity score of 0, 20 with a score of 1, 62 with a score of 2, and 56 with a score of 3. Typically, each subject provided two finger tapping test videos, one for each hand, but not all subjects had both videos. This dataset did not contain any subject with the worst possible severity score of 4.

Subjects were early in the disease progression, with an average disease duration of 20.8 ± 17.9 months [28].

### B. Video Processing

Fig. 2 illustrates the hand tracking results provided by our video processing pipeline. The figure shows three keyframes during the movement cycle, including the thumb and index fingers fully closed, fully extended, and fully closed again. The figure shows the landmarks positions provided by Google’s MediaPipe, the vectors formed by joining the base of the hand and the tip of the thumb and index fingers and estimated the angular distance between the vectors.

**Fig. 4.**
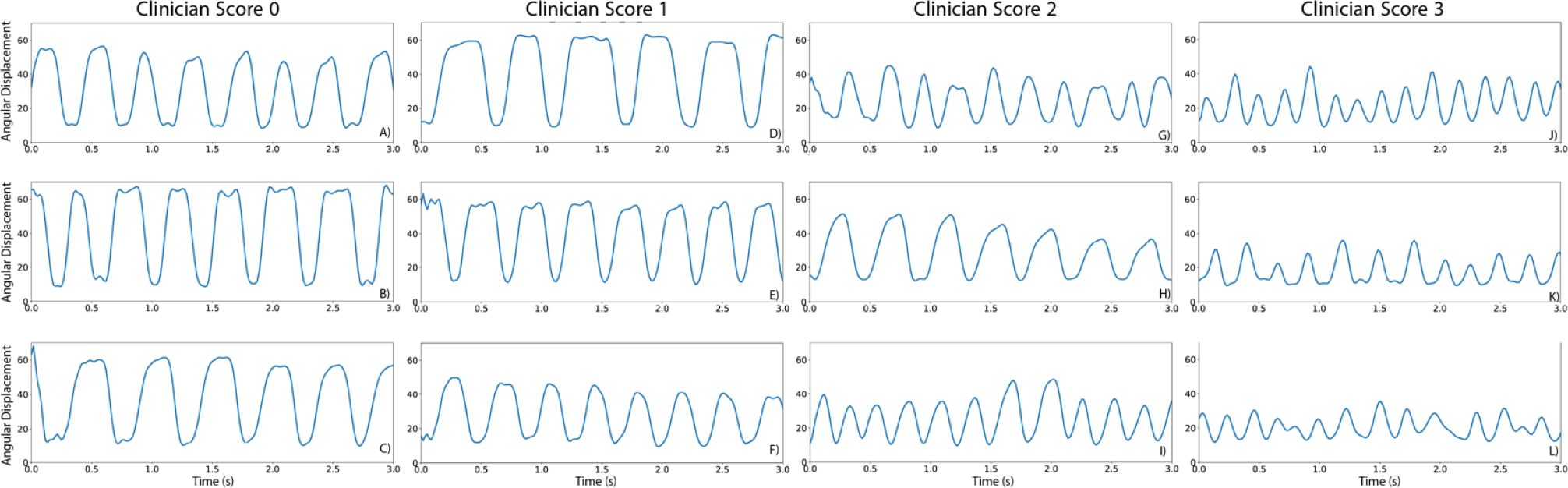
Angular distance between the index and thumb derived from videos of subjects performing the finger tapping test using ML algorithms for hand pose estimation. A, B and C) Subjects that received a clinician score of 0, indicating normal movement. D, E, and F) Subjects that received a clinician score of 1, indicating slight motor symptoms. G, H, and I) Subjects that received a clinician score of 2, indicating mild motor symptoms. And J, K, and L) Subjects that received a clinician score of 3, indicating moderate motor symptoms.

Fig. 4 presents a 3s segment of the angular displacement computed from twelve videos in our dataset. Fig. 4 A, B, and C show the angular displacement for subjects that received a severity score of 0 by a trained clinician. In this case, the subjects fully opened and closed their fingers in each movement cycle. Fig. 4 D, E, and F show the angular displacement for subjects that received a severity score of 1. In this case, the movements are similar to those observed for scores of 0. Fig. 4 G, H, and I show the angular displacement for subjects that received a severity score of 2. In this case, displacement signals show increased cycle-to-cycle movement variability, and one of the subjects demonstrated a continuous decrease of amplitude during the tasks, a phenomenon known as *sequence effect*. Finally, Fig. 4 J, K, and L show the angular distance for subjects that received a clinical score of 3. In this case, subjects demonstrated small movement amplitude with large cycle-to-cycle variability.

### C. Classification Results

1. *Multiclass Classification:* A multiclass logistic regression model was trained to predict the clinician-provided severity score for the Finger tapping task based on video-based kinematic features, age, and sex. The feature selection procedure identified six key features critical for classification, including Mean Amplitude, Mean Speed, Mean Opening Speed, Mean Closing Speed, Mean Cycle Duration, and CV of Cycle Duration. Using these six features, the model demonstrated an average AUC of the Recall-Precision curve of 0.39 and an average f1-score of 0.42. The model accuracy was 30% for score of 0, 67% for score of 1, 21% for score of 2, and 59% for score of 3.
2. *Ordinal Binary Classification:* Three binary logistic regression models were developed to predict the clinician-provided severity score for the Finger tapping test based on video-based measures, age, and sex.
  - Score [0] vs. Scores [1, 2, 3]:
    The feature selection procedure identified three key features critical for classification, including Mean Speed, Mean Opening Speed, and Mean Closing Speed. Using these three features, the model demonstrated an AUC of the Recall-Precision curve of 0.94 and a f1-score of 0.87. The model accuracy was 69% for score of 0 and 86% for score 1 to 3.
  - Score [0, 1] vs. scores [2, 3]:
    The feature selection procedure identified five key features critical for classification, including Mean Amplitude, Mean Opening Speed, CV Closing Speed, Mean Cycle Duration, and CV of Cycle Duration. Using these five features, the model demonstrated an AUC of the Recall-Precision curve of 0.85 and a f1-score of 0.84. The model accuracy was 60% for score of 1 or 2 and 89% for score of 2 or 3.
  - Score [0,1,2] vs. scores [3]:
    The feature selection procedure identified five key features critical for classification, including Mean Closing Speed, Mean Opening Speed, CV Amplitude, CV Opening Speed, and sex. Using these five features, the model demonstrated an AUC of the Recall-Precision curve of 0.56 and a f1-score of 0.61. The model accuracy was 84% for score of 1,2, or 3 and 60% for score of 3.
3. *Tiered Binary Classification:* Three binary logistic regression models were developed to predict the clinician-provided severity score for the Finger tapping test based on video-based measures, age, and sex.
  - HC vs. PD The feature selection procedure identified five key features critical for classification, including Mean Speed, Mean Closing Speed, Mean Cycle Duration, Amplitude Decay, and age. Using these five features, the model demonstrated an AUC of the Recall-Precision curve of 0.97 and a f1-score of 0.91. The model accuracy was 85% for HC and 88% for PD.
  - Score [1] vs. Scores [2, 3]:
    The feature selection procedure identified six key features critical for classification, including Mean Amplitude, Mean Speed, Mean Opening Speed, CV Closing Speed, Amplitude Decay, and CV of Cycle Duration. Using these five features, the model demonstrated an AUC of the Recall-Precision curve of 0.97 and a f1-score of 0.88. The model accuracy was 100% for score of 1 and 77% for score of [2] and [3]
  - Score [2] vs. Score [3]:
    The feature selection procedure identified four key features critical for classification, including CV Opening Speed, Range of Cycle Duration, Amplitude Decay, and age. Using these four features, the model demonstrated an AUC of the Recall-Precision curve of 0.89 and a f1-score of 0.81. The model accuracy was 84% for score of 2 and 80% for score of 3.

### D. Differences Between Groups

Table II presents the group differences for the video-based movement features as a function of clinician assigned severity score. Ten features demonstrated significant difference between groups, including Mean of Amplitude, Speed, and Cycle Duration, CV of Amplitude, Speed, Opening Speed, Closing Speed, and Cycle Duration, range of Cycle Duration, and Amplitude Decay.

**Fig. 5.**
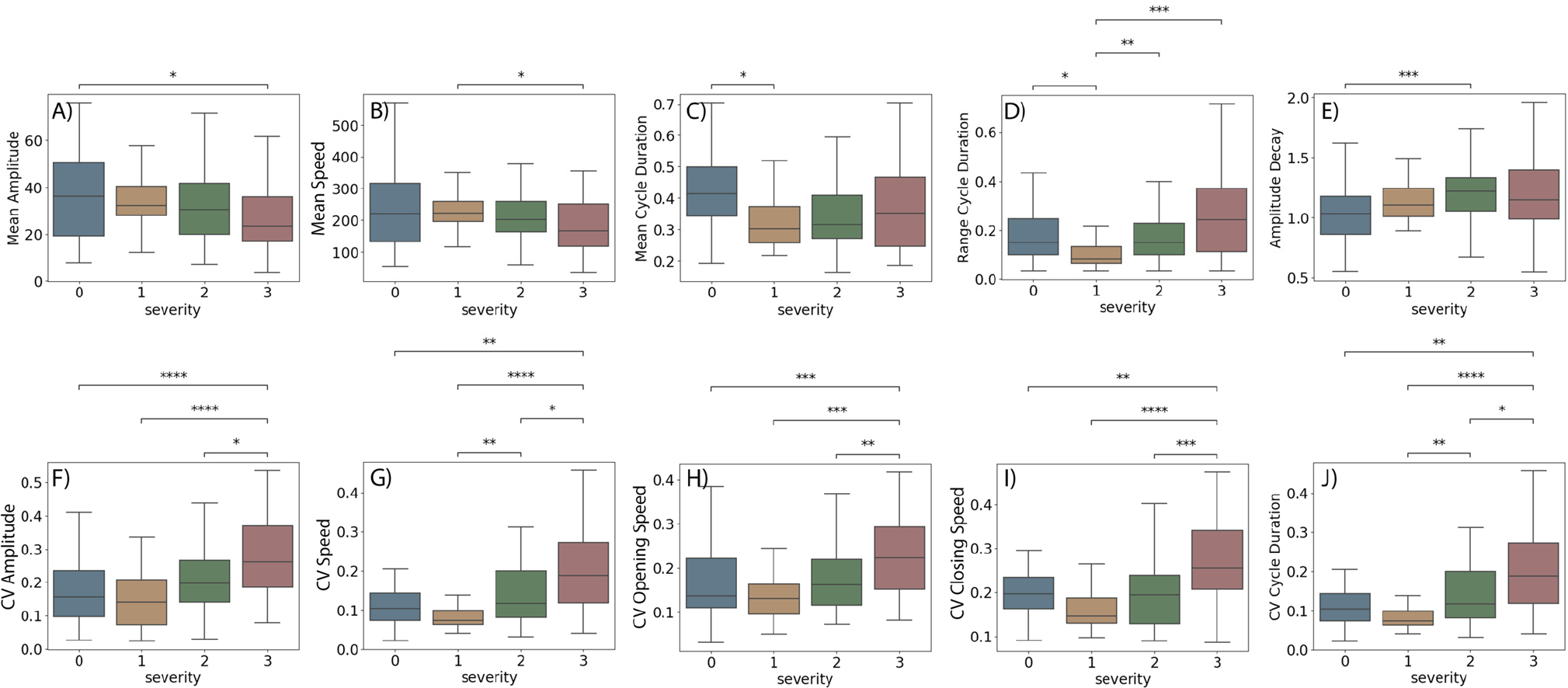
Groups differences for the movement features identified by the ANOVA analysis to be significant different between groups. A) Mean Amplitude, B) Mean RMS Velocity, C) Mean Cycle Duration, D) Movement Rate, E) Amplitude Decay, F) CV Amplitude, G) CV RMS Velocity, H) CV Average Opening Speed, I) CV Average Closing Speed, and J) CV Cycle Duration. p-values were corrected for multiple comparisons using Bonferroni correction. p-value legend, *: 1.00e-02 < p-value <=5.00e-02, **: 1.00e-03 < p-value <= 1.00e-02, ***: 1.00e-04 < p-value <= 1.00e-03, ****: p-value <= 1.00e-04.

Finally, Fig. 5 presents the results of the Tukey’s Honest Significant Difference post-hoc test with Bonferroni correction between different pairs of severity scores for all the video-based movement features that demonstrated significant difference between groups. Next, we will discuss the video-based measures that differ between severity scores.

- Score [0] vs. Score [1]
  Mean Cycle Duration, and Range of Cycle Duration were significant different between persons with severity Score of 0 and 1.
- Score [0] vs. Score [2]
  Amplitude Decay was significant different between persons with severity Score of 0 and 2.
- Score [0] vs. Score [3]
  Mean Amplitude, CV of Amplitude, CV Speed, CV of Opening Speed, CV of closing Speed, and CV of Cycle Duration were significant different between persons with severity Score of 0 and 3.
- Score [1] vs. Score [2]
  Range of Cycle Duration, CV of Speed, and CV of Cycle Duration were significant different between persons with severity Score of 1 and 2.
- Score [1] vs. Score [3]
  Mean Amplitude, Mean Speed, CV of Amplitude, CV of Speed, CV of Opening Speed, CV of Closing Speed, Range of Cycle Duration, and CV of Cycle Duration were significant different between persons with severity Score of 1 and 3.
- Score [2] vs. Score [3]
  CV of Amplitude, CV of Speed, CV of Opening Speed, CV of Closing Speed, and CV of Cycle Duration were significant different between persons with severity Score of 2 and 3.

## IV. Discussion

The Finger Tapping Test is commonly used to assess bradykinesia in the upper extremities. This motor evaluation is an important component of several standardized clinical test used to assess upper limbs motor function in different neurological conditions, including PD and other forms of parkinsonism [2], [29], [30]. Individuals with PD and other neurological diseases demonstrate diminished movement speed and amplitude with increased movement hesitations/halts when compared to healthy controls [31]. Some patients also demonstrate increased movement variability and a progressive reduction in movement amplitude during the repetitive tapping movement (sequence effect) [32], [33]. Several studies have shown that is possible to quantify kinematic features related bradykinesia such as amplitude, speed, and rate from videos of the Finger Tapping videos using ML algorithms [8], [10], [11], [15], [16], [17], [18], [19], [34], [35]. In this study, we showed that other, non-traditional kinematic features such as opening and closing movement speed, amplitude decay, and measures related to movement and timing variability could also be quantified from videos. Our results show that these kinematic features are significantly different as a function of motor symptoms severity, and they are key for video-based prediction of motor symptoms severity. Previous studies that collected data using accelerometers attached to the fingers or motion capture systems found similar results [33], [36], [37].

The ability to automatically predict the severity of PD from videos could revolutionize PD management by facilitating monitoring and quantifying motor symptoms severity from simple video recordings. Currently, assessment of motor function in PD requires a neurologist with expertise in movement disorders, and assessments occur scarcely due to the limited availability of clinical experts. Furthermore, clinician assessments are bound by a 5-point Likert scale that might not be sensitive to subtle, yet significant alterations in motor function [2], [38], [39]. A commonly cited reason for failure of PD disease altering clinical trials is the lack of perfect progression endpoints in PD, heterogeneity among subjects is significant to the point that PD may be more appropriately called a syndrome with multiple causes [1]. A known floor effect, intra- and inter-rater reliability issues, and great susceptibility to symptomatic treatment related variability are additional concerns about using the MDS-UPDRS to monitor progression of motor symptoms [40]. In contrast, video-based assessments can help monitor disease progression or treatment response on regular basis, and the quantitative nature of video derived kinematic features can help to quantify disease severity as a continuous rather than a discrete variable, potentially increasing the scale’s sensitivity.

Previous studies have used kinematic features derived from finger tapping videos using ML algorithms for hand pose estimation and tracking to automatically predict disease severity. Most studies employ a multiclass classification approach, where movement features are used as part of a multiclass classification model to predict disease severity (a score from 0 to 4) [15], [16], [17], [18], [19]. Using this approach, we observed a moderate classification performance, the average test set accuracy was 45%. Our results align well with the results recently published by Islam et al. [17].

These results highlight the limitations of using a single multiclass model for predicting disease severity in PD. Such a model assumes that the movement features change proportionally across disease severity. However, this assumption does not align with reality. Our results from Table II show that most of the video-based kinematic features differ significantly between groups, but when analyzing the differences between severity scores, we observed that the features that differed between groups with the lower scores (Mean Cycle Duration, Rate, and Amplitude Decay) were different from the features that differed between groups with the highest scores (all the variability measures). This observation agrees with our hypothesis that the features that mark disease severity differ as the disease progresses. Therefore, it may be more effective to use a combination of models or a multi-stage modeling approach that accounts for different feature sets at various severity levels, rather than relying on a single multiclass model.

Morinan et al. proposed an alternative to the multiclass classification approach [8]. Their methodology, known as ‘Ordinal Binary Classification’, employs a series of binary classifiers designed to reflect the ordinal nature of disease severity. Using this approach, we observed a moderate to good classification performance. The average test set accuracy was 74%. Our results align well with published results.

In this study, we introduced a new classification approach based on our hypothesis that features able to the characterize disease severity differ as the disease progresses. Our methodology, called ‘Tiered Binary Classification’, employs a series of binary classification models trained to identify different levels of disease severity. The first model classifies healthy controls vs. persons with PD, the second model classified scores of 1 vs. scores of 2 and 3, and the final model classified scores of 2 vs. scores of 3. The average test set accuracy was 86%.

The multiclass, ordinal binary, and tiered binary classification approaches cannot be compared directly. However, in our dataset, all videos with a clinical severity score of 0 belonged to HC, thus, it is possible to directly compare the ability of these three approaches to identify HC from patients. The multiclass classification approach yielded an accuracy of 30% when classifying videos with a severity score of 0. In the case of the ordinal binary classification approach, the accuracy was 69%. Finally, for our proposed tiered binary classification approach, the accuracy was 85%.

In our proposed classification approach, a video undergoes an analysis by an initial model that determines whether the subject is a healthy individual or a person with PD. If PD is detected, the video is further assessed by a series of specialized models to evaluate disease severity based on motor performance. The second model discerns whether to assign a severity score of 1 or higher, while the third model distinguishes between scores of 2 and 3. Due to the absence of subjects with a severity score of 4 in our dataset, we did not develop a model for this stage. Each model utilizes distinct movement features identified through a data-driven approach, reinforcing our hypothesis that different movement characteristics become more indicative of disease severity at progressive stages of the disease. Our findings indicate that features such as movement amplitude, speed, cycle duration, and amplitude decay are particularly telling for distinguishing healthy individuals from those with mild or moderate PD. In contrast, features concerning movement and timing variability are more discriminative for differentiating between mild and moderate severity levels.

Finally, our findings indicate that traditional movement features such as amplitude and speed consistently decreased with increasing disease severity, as depicted in Fig. 5 A and B. In contrast, other movement characteristics exhibit non-linear trends. Notably, the mean cycle duration was substantially longer for severity score 0 compared to score 1, then increased slightly for severity scores 2 and 3. This pattern is attributable to the fact that while subjects at severity score1 demonstrated a reduced movement amplitude compared to score 0 (32.86±12.95 deg vs. 36.81±18.96 deg), their movement velocity remained similar (230.82±70.67 deg/s vs. 231.11±125.4 deg/s), resulting in a shorter time to complete a movement cycle. Moreover, amplitude decay escalated from severity scores 0 to 1, then again from 1 to 2, but diminished from 2 to 3, potentially due to the reduced movement amplitude at score 3, which hinders the detection of amplitude changes within the task in patients with more severe motor symptoms. As shown in Fig. 5 G – J, the variability measures decreased from severity scores 0 to 1 but then rose significantly with further disease advancement. The initial reduction in variability could be due to compensatory mechanisms that attempt to maintain functional movements. However, as the disease advances these compensatory strategies cannot be maintained, leading to an increase in movement irregularities and variability as the neuromuscular system’s ability to control movement deteriorates. These trends underscore the complexity of PD progression and highlight the potential for these nuanced features to improve the accuracy of severity assessments.

Our study includes several limitations. First, the disease accuracy was based on a single clinician assessment of the Finger Tapping test. The assessment occurred live while the subjects were performing the test. It is known that multiple raters might provide a different score for the same subject, especially for low scores, indicating that a score provided by a single rater might not reflect the true severity of motor symptoms [2], [38], [39]. To make a fairer comparison between clinician-provided and automatic scores, future studies must employ multiple raters and identify their agreement. Second, we included a limited number of participants, and our dataset is unbalanced, with more videos with a score of 2 and 3 than 0 and 1. We employed the SMOTE algorithm to balance our dataset [22], but such imbalance might still affect our models. Future studies should develop balanced datasets to avoid this issue altogether. Third, our patient’s dataset only included persons with PD with slight, mild, and moderate motor signs. We did not include patients with very subtle or severe motor symptoms. Consequently, the observations made in this study might not translate to early-stage or late-stage PD. Future studies must include de-novo patients with only subtle motor alterations and patients in the later stages if the disease to get a full picture of the disease progression. Finally, during the recording sessions, a clinician was present to guide the participants during the execution of the task. Because of this, our data has little variability regarding hand position, so that we could not study the effects of hand position in the hand pose estimation results.

## V. Conclusion

This study offers two significant contributions. First, we introduced an automated approach to objectively quantify kinematic features related to bradykinesia from videos of the Finger Tapping task. Similar to previous studies, our method quantifies traditional kinematic features such as movement amplitude, speed, and amplitude decay, which vary linearly with motor symptom severity. Additionally, we quantified non-traditional kinematic features, including cycle duration, opening and closing speeds, and variability measures. These features are challenging to estimate visually and are not commonly used by clinicians when assessing motor symptom severity. Our results demonstrate that these non-traditional kinematic features can be accurately estimated from videos, exhibit significant but non-linear changes with motor symptom severity, and enhance the video-based prediction of disease severity.

The second contribution of this study involves the introduction of a novel tiered classification method that utilizes different kinematic features based on the severity of the disease. This approach contrasts with traditional methods that rely on a consistent set of features across all severity levels. The tiered classification method mirrors the clinical practice of assessing patients, where clinicians focus on different motor aspects to assign increasing scores. Moreover, our approach demonstrated superior accuracy in predicting disease severity and distinguishing between different severity levels compared to existing methods. This technique enhances the ability to provide a continuous and detailed assessment of motor symptoms, potentially leading to significant improvements in PD management and the evaluation of treatment efficacy.

Our results demonstrate the feasibility of video-based assessment for accurate prediction of motor symptoms severity in PD. In our future work, we will employ the tiered binary classification strategy proposed here with videos recorded at home without the direct guidance of a clinician.

## Figures and Tables

**TABLE I:** Demographic Information

	Controls	Persons with PD

N (female | male)	24 (12 | 12)	66 (25 | 41)
Age (years) mean ± std [range]	62.7 ± 8.1 [50 – 77]	63.5± 7.8 [44 – 77]
Disease Duration in months mean ± std [range]		20.8 ± 17.9 [0 – 74]
H&Y mean ± std [range]		1.8 ± 0.5 [1 – 3]
UPDRS-III mean ± std [range]		32.6+ 10.6 [12 – 60]
UPDRS		
Finger Tap Right mean ± std [range]		1.9 ± 0.9 [0 – 3]
UPDRS		
Finger Tap Left mean ± std [range]		2.0 ± 0.9 [0 – 3]

H&Y -> Hoehn and Yahr Scale.

UPDRS-III -> Unified Parkinson’s Disease Rating Scale.

**TABLE II:** Group Differences for the Video-Based Movement Features as a Function of Clinician Assigned Score

Feature	Score 0 (N=42)	Score 1 (N=20)	Score 2 (N=62)	Score 3 (N=56)	ANOVA	
					F	P

Mean Amplitude	36.81±18.96	32.86±12.95	31.04±26.87	26.87±87.99	3.55	<0.05
Mean Speed	231.11±125.4	230.82±70.67	209.77±76.29	184.06±84.32	2.58	<0.05
Mean Opening Speed	185.23±101.2	186.04±60.34	163.72±63.91	147.40±70.04	2.26	0.056
Mean Closing Speed	187.73±108.4	197.93±63.44	183.96±67.43	159.67±72.33	1.73	0.161
CV Amplitude	0.17±0.10	0.15±0.10	0.21±0.10	0.28±0.12	10.43	≪0.05
CV Speed	0.17±0.09	0.14±0.06	0.18±0.07	0.23±0.08	8.57	≪0.05
CV Opening Speed	0.20±0.09	0.16±0.06	0.20±0.08	0.27±0.09	10.30	≪0.05
CV Closing Speed	0.21±0.10	0.18±0.06	0.23±0.08	0.29±0.10	7.20	≪0.05
Mean Cycle Duration	0.44±0.17	0.33±0.12	0.37±0.16	0.37±0.16	2.58	<0.05
CV Cycle Duration	0.12±0.09	0.08±0.02	0.14±0.09	0.24±0.16	8.47	≪0.05
Range of Cycle Duration	0.19±0.17	0.09±0.05	0.20±0.17	0.36±0.57	3.98	<0.05
Rate	2.82±1.00	3.20±0.82	3.08±0.98	3.09±1.13	2.48	0.062
Amplitude Decay	1.02±0.10	1.13±0.16	1.23±0.32	1.20±1.36	2.77	<0.05

## References

[1] Devos D, Hirsch E, and Wyse R, “Seven solutions for neuroprotection in Parkinson’s disease,” Movement Disorders, vol. 36, no. 2, pp. 306–316, Feb. 2021, doi: 10.1002/mds.28379. 33184908

[2] Goetz CG , “Movement disorder society-sponsored revision of the unified Parkinson’s disease rating scale (MDS-UPDRS): Scale presentation and clinimetric testing results,” Movement Disorders, vol. 23, no. 15, pp. 2129–2170, Nov. 2008, doi: 10.1002/mds.22340. 19025984

[3] Postuma RB , “MDS clinical diagnostic criteria for Parkinson’s disease,” Movement Disorders, vol. 30, no. 12, pp. 1591–1601, Oct. 2015, doi: 10.1002/mds.26424. 26474316

[4] Siderowf A, McDermott M, Kieburtz K, Blindauer K, Plumb S, and Shoulson I, “Test–retest reliability of the unified Parkinson’s disease rating scale in patients with early Parkinson’s disease: Results from a multicenter clinical trial,” Movement Disorders, vol. 17, no. 4, pp. 758–763, Jul. 2002, doi: 10.1002/mds.10011. 12210871

[5] Tosin MHS, Stebbins GT, Comella C, Patterson CG, and Hall DA, “Does MDS-UPDRS provide greater sensitivity to mild disease than UPDRS in de novo Parkinson’s disease?” Movement Disorders Clin. Pract, vol. 8, no. 7, pp. 1092–1099, Sep. 2021, doi: 10.1002/mdc3.13329. PMC8485601 34631945

[6] de Deus Fonticoba T, García DS, and Arribí MM, “Inter-rater variability in motor function assessment in Parkinson’s disease between experts in movement disorders and nurses specialising in PD management,” (English Edition), Neurología, vol. 34, no. 8, pp. 520–526, Oct. 2019, doi: 10.1016/j.nrleng.2017.03.006. 28549752

[7] Post B, Merkus MP, de Bie RMA, de Haan RJ, and Speelman JD, “Unified Parkinson’s disease rating scale motor examination: Are ratings of nurses, residents in neurology, and movement disorders specialists interchangeable?” Movement Disorders, vol. 20, no. 12, pp. 1577–1584, Dec. 2005, doi: 10.1002/mds.20640. 16116612

[8] Morinan G , “Computer vision quantification of whole-body Parkinsonian bradykinesia using a large multi-site population,” NPJ Parkinson’s Disease, vol. 9, no. 1, Jan. 2023, Art. no. 1, doi: 10.1038/s41531-023-00454-8. PMC9883391 36707523

[9] Sibley KG, Girges C, Hoque E, and Foltynie T, “Video-based analyses of Parkinson’s disease severity: A brief review,” J. Parkinson’s Disease, vol. 11, no. s1, pp. S83–S93, Jul. 2021, doi: 10.3233/jpd-202402. 33682727 PMC8385513

[10] Acevedo Trebbau GT, Bandini A, and Guarin DL, “Video-based hand pose estimation for remote assessment of bradykinesia in Parkinson’s disease,” in Predictive Intelligence in Medicine (Lecture Notes in Computer Science), Rekik I, Adeli E, Park SH, Cintas C, and Zamzmi G, Eds. Cham, Switzerland: Springer, 2023, pp. 241–252, doi: 10.1007/978-3-031-46005-0_21.

[11] Monje MHG , “Remote evaluation of Parkinson’s disease using a conventional webcam and artificial intelligence,” Frontiers Neurol., vol. 12, Dec. 2021, Art. no. 742654, doi: 10.3389/fneur.2021.742654. PMC8733479 35002915

[12] Arpin DJ , “Diffusion magnetic resonance imaging detects progression in Parkinson’s disease: A placebo-controlled trial of rasagiline,” Movement Disorders, vol. 37, no. 2, pp. 325–333, Feb. 2022, doi: 10.1002/mds.28838. 34724257 PMC9019575

[13] Hughes AJ, Daniel SE, Kilford L, and Lees AJ, “Accuracy of clinical diagnosis of idiopathic Parkinson’s disease: A clinico-pathological study of 100 cases,” J. Neurol., Neurosurgery Psychiatry, vol. 55, no. 3, pp. 181–184, Mar. 1992, doi: 10.1136/jnnp.55.3.181. PMC1014720 1564476

[14] Zhang F , “MediaPipe hands: On-device real-time hand tracking,” 2020, arXiv:2006.10214.

[15] Li H, Shao X, Zhang C, and Qian X, “Automated assessment of Parkinsonian finger-tapping tests through a vision-based fine-grained classification model,” Neurocomputing, vol. 441, pp. 260–271, Jun. 2021, doi: 10.1016/j.neucom.2021.02.011.

[16] Guo Z , “Vision-based finger tapping test in patients with Parkinson’s disease via spatial–temporal 3D hand pose estimation,” IEEE J. Biomed. Health Informat, vol. 26, no. 8, pp. 3848–3859, Aug. 2022, doi: 10.1109/JBHI.2022.3162386. 35349459

[17] Islam MS , “Using AI to measure Parkinson’s disease severity at home,” NPJ Digit. Med, vol. 6, no. 1, Aug. 2023, Art. no. 1, doi: 10.1038/s41746-023-00905-9. 37608206 PMC10444879

[18] Yang N , “Automatic detection pipeline for accessing the motor severity of Parkinson’s disease in finger tapping and postural stability,” IEEE Access, vol. 10, pp. 66961–66973, 2022, doi: 10.1109/ACCESS.2022.3183232.

[19] Li Z, Lu K, Cai M, Liu X, Wang Y, and Yang J, “An automatic evaluation method for Parkinson’s dyskinesia using finger tapping video for small samples,” J. Med. Biol. Eng, vol. 42, no. 3, pp. 351–363, Jun. 2022, doi: 10.1007/s40846-022-00701-y.

[20] Guarin DL, Taati B, Abrahao A, Zinman L, and Yunusova Y, “Video-based facial movement analysis in the assessment of bulbar amyotrophic lateral sclerosis: Clinical validation,” J. Speech, Lang., Hearing Res., vol. 65, no. 12, pp. 4667–4678, Dec. 2022, doi: 10.1044/2022_jslhr-22–00072. PMC9940890 36367528

[21] Pedregosa F , “Scikit-learn: Machine learning in Python,” J. Mach. Learn. Res, vol. 12, pp. 2825–2830, Nov. 2011.

[22] Chawla NV, Bowyer KW, Hall LO, and Kegelmeyer WP, “SMOTE: Synthetic minority over-sampling technique,” J. Artif. Intell. Res, vol. 16, pp. 321–357, Jun. 2002, doi: 10.1613/jair.953.

[23] Alghamdi M, Al-Mallah M, Keteyian S, Brawner C, Ehrman J, and Sakr S, “Predicting diabetes mellitus using SMOTE and ensemble machine learning approach: The Henry Ford ExercIse testing (FIT) project,” PLoS ONE, vol. 12, no. 7, Jul. 2017, Art. no. e0179805, doi: 10.1371/journal.pone.0179805. PMC5524285 28738059

[24] Hussain L, Lone KJ, Awan IA, Abbasi AA, and Pirzada J-U-R, “Detecting congestive heart failure by extracting multimodal features with synthetic minority oversampling technique (SMOTE) for imbalanced data using robust machine learning techniques,” Waves Random Complex Media, vol. 32, no. 3, pp. 1079–1102, May 2022, doi: 10.1080/17455030.2020.1810364.

[25] Ishaq A , “Improving the prediction of heart failure Patients’ survival using SMOTE and effective data mining techniques,” IEEE Access, vol. 9, pp. 39707–39716, 2021, doi: 10.1109/ACCESS.2021.3064084.

[26] Vallat R, “Pingouin: Statistics in Python,” J. Open Source Softw, vol. 3, no. 31, p. 1026, Nov. 2018, doi: 10.21105/JOSS.01026.

[27] Virtanen P , “SciPy 1.0: Fundamental algorithms for scientific computing in Python,” Nature Methods, vol. 17, no. 3, pp. 261–272, Mar. 2020, doi: 10.1038/s41592-019-0686-2. 32015543 PMC7056644

[28] Skorvanek M , “Differences in MDS-UPDRS scores based on Hoehn and Yahr stage and disease duration,” Movement Disorders Clin. Pract, vol. 4, no. 4, pp. 536–544, Jul. 2017, doi: 10.1002/mdc3.12476. PMC6174385 30363418

[29] Hall DA , “Clinimetric properties of the fragile X-associated tremor ataxia syndrome rating scale,” Movement Disorders Clin. Pract, vol. 6, no. 2, pp. 120–124, Jan. 2019, doi: 10.1002/mdc3.12708. PMC6384171 30838310

[30] Sabari JS, Woodbury M, and Velozo CA, “Rasch analysis of a new hierarchical scoring system for evaluating hand function on the motor assessment scale for stroke,” Stroke Res. Treatment, vol. 2014, Aug. 2014, Art. no. 730298, doi: 10.1155/2014/730298. PMC4142312 25177513

[31] Arias P, Robles-García V, Espinosa N, Corral Y, and Cudeiro J, “Validity of the finger tapping test in Parkinson’s disease, elderly and young healthy subjects: Is there a role for central fatigue?” Clin. Neurophysiol, vol. 123, no. 10, pp. 2034–2041, Oct. 2012, doi: 10.1016/j.clinph.2012.04.001. 22560636

[32] Djurić -Jovič ić M , “Finger tapping analysis in patients with Parkinson’s disease and atypical parkinsonism,” J. Clin. Neurosci, vol. 30, pp. 49–55, Aug. 2016, doi: 10.1016/j.jocn.2015.10.053. 27343040

[33] Krupič ka R , “Instrumental analysis of finger tapping reveals a novel early biomarker of parkinsonism in idiopathic rapid eye movement sleep behaviour disorder,” Sleep Med, vol. 75, pp. 45–49, Nov. 2020, doi: 10.1016/j.sleep.2020.07.019. 32853917

[34] Khan T, Nyholm D, Westin J, and Dougherty M, “A computer vision framework for finger-tapping evaluation in Parkinson’s disease,” Artif. Intell. Med, vol. 60, no. 1, pp. 27–40, Jan. 2014, doi: 10.1016/j.artmed.2013.11.004. 24332155

[35] Yu T, Park KW, McKeown MJ, and Wang ZJ, “Clinically informed automated assessment of finger tapping videos in Parkinson’s disease,” Sensors, vol. 23, no. 22, p. 9149, Nov. 2023, doi: 10.3390/s23229149. 38005535 PMC10674854

[36] Cochen De Cock V , “Rhythm disturbances as a potential early marker of Parkinson’s disease in idiopathic REM sleep behavior disorder,” Ann. Clin. Transl. Neurol, vol. 7, no. 3, pp. 280–287, Mar. 2020, doi: 10.1002/acn3.50982. 32059086 PMC7085999

[37] Yokoe M, Okuno R, Hamasaki T, Kurachi Y, Akazawa K, and Sakoda S, “Opening velocity, a novel parameter, for finger tapping test in patients with Parkinson’s disease,” Parkinsonism Rel. Disorders, vol. 15, no. 6, pp. 440–444, Jul. 2009, doi: 10.1016/j.parkreldis.2008.11.003. 19103505

[38] Heldman DA , “The modified bradykinesia rating scale for Parkinson’s disease: Reliability and comparison with kinematic measures,” Movement Disorders, vol. 26, no. 10, pp. 1859–1863, Aug. 2011, doi: 10.1002/mds.23740. 21538531 PMC3324112

[39] Ramaker C, Marinus J, Stiggelbout AM, and van Hilten BJ, “Systematic evaluation of rating scales for impairment and disability in Parkinson’s disease,” Movement Disorders, vol. 17, no. 5, pp. 867–876, Sep. 2002, doi: 10.1002/mds.10248. 12360535

[40] Trifonova OP , “Parkinson’s disease: Available clinical and promising omics tests for diagnostics, disease risk assessment, and pharmacotherapy personalization,” Diagnostics, vol. 10, no. 5, p. 339, May 2020, doi: 10.3390/diagnostics10050339. 32466249 PMC7277996

